# Log-Less Metadata Management on Metadata Server for Parallel File Systems

**DOI:** 10.1155/2014/813521

**Published:** 2014-04-27

**Authors:** Jianwei Liao, Guoqiang Xiao, Xiaoning Peng

**Affiliations:** ^1^College of Computer and Information Science, Southwest University of China, Beibei, Chongqing 400715, China; ^2^HuaiHua College of Computer Science and Technology, Huaihua, Hunan 418008, China

## Abstract

This paper presents a novel metadata management mechanism on the metadata server (MDS) for parallel and distributed file systems. In this technique, the client file system backs up the sent metadata requests, which have been handled by the metadata server, so that the MDS does not need to log metadata changes to nonvolatile storage for achieving highly available metadata service, as well as better performance improvement in metadata processing. As the client file system backs up certain sent metadata requests in its memory, the overhead for handling these backup requests is much smaller than that brought by the metadata server, while it adopts logging or journaling to yield highly available metadata service. The experimental results show that this newly proposed mechanism can significantly improve the speed of metadata processing and render a better I/O data throughput, in contrast to conventional metadata management schemes, that is, logging or journaling on MDS. Besides, a complete metadata recovery can be achieved by replaying the backup logs cached by all involved clients, when the metadata server has crashed or gone into nonoperational state exceptionally.

## 1. Introduction

Distributed and parallel file systems employ multiple parallel I/O devices by striping file data across the I/O nodes and then through using high aggregate bandwidth to meet the growing I/O requirements of parallel scientific applications [1, 2]. In addition, decoupling file's metadata from read and write operations has been proven to be an effective strategy to improve the concurrency in the parallel file systems, since the operations on metadata and real file data could be processed in parallel [3]. Generally speaking, in a parallel file system, a client file system (client) communicates with the active metadata server (MDS), which manages all properties of the whole file system, to get the permission to operate on the file and file's layout information that indicates the locations of storage servers (OSTs), on which the stripes belonging to the target file are stored. Then the client accesses the corresponding OSTs, which handles management of actual file data on the storage devices, to perform the real file I/O operations after parsing the file's layout information.

The metadata server (MDS) plays an intermediary role in a parallel file system, and the metadata is essential to the whole file system. Both interruption of the metadata service and inconsistent metadata may lead the entire file system to become unavailable [4]. Most of the parallel file systems, such as Gfarm [5], Ceph [6], and GFS [7], employ logging or journaling metadata updates on the MDS; therefore a complete and up-to-date metadata snapshot can be yielded by resorting to the logs or journals, which have been committed to the nonvolatile storage devices, when the former active MDS has crashed. Without doubts, however, logging all metadata updates slows down the speed of metadata processing on MDS, which is because the metadata response cannot be sent until the corresponding metadata update has been committed to the nonvolatile storage devices.

In order to eliminate the negative effect of logging metadata changes conducted by the MDS, based on our previous work [8, 9] this paper proposes a log-less metadata management mechanism on the metadata server. To put it from another angle, the metadata server does not create any logs to record metadata changes. In the newly proposed mechanism, all clients back up metadata requests that incorporated with the required metadata response sent by the MDS till the associated metadata changes have been committed to the nonvolatile storage. Once the former MDS has crashed, all involved clients are supposed to resend the backup metadata requests, so that the current MDS can restore all lost metadata changes by replaying these requests. Since every metadata request is backed up by several clients in this newly proposed mechanism, the complete and up-to-date metadata recovery is still achievable even though one or more clients failed to resend the backup requests. As a result, the MDS is freed from logging metadata updates to nonvolatile storage; thus, the speed of metadata processing can be improved to a great extent, which is the main goal of this newly proposed metadata management mechanism.

This paper is organized as follows: we first present the design and implementation of the log-less metadata management mechanism in Section 2; next, the evaluation experiments and results are demonstrated in Section 3; then, Section 4 describes the related work about metadata management for ensuring metadata consistency; finally, we make concluding remarks.

## 2. Design and Implementation

### 2.1. The Interactivity between Client and MDS

**Figure 1:**
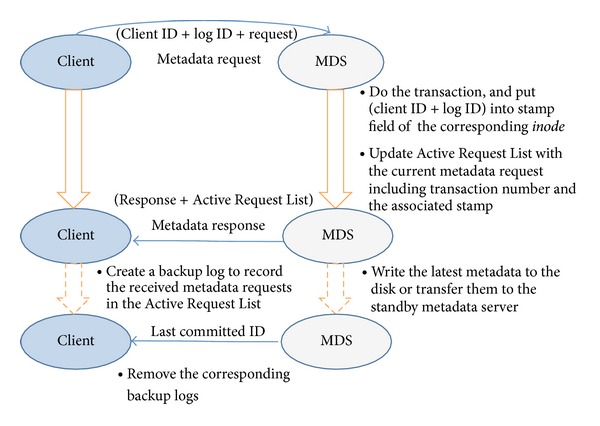
The interaction between client and MDS.

Figure 1 illustrates the normal case of interactivity between the client and the MDS in the log-less metadata management mechanism. The client sends the request with a log ID and client ID; after the transaction involving the metadata request, the MDS reads the stamp field of the associated* inode* (a data structure in the file systems that stores all the information about the file system objects, such as file and directory, but without data and name) and sends it along with the metadata response to the client; finally, it puts the client ID and log ID into the stamp field of the corresponding* inode* to indicate the last metadata request related to the* inode* and updates Active Request List with the current metadata request including the sequential number generated by the MDS (i.e., the corresponding transaction number) and the relevant stamp. The client, after receiving the response replied by MDS, creates a backup log in the memory to record all metadata requests (the requests in the Active Request List) sent by the MDS with the response.

The backup logs can be removed from memory after the associated metadata changes have been written to the disk. Once the active MDS has crashed, the rebooted MDS or the standby MDS can restore the lost metadata by reexecuting the backup requests stored on the client side.

### 2.2. Client Caching Metadata Requests

As we mentioned in Section 2.1, the client file system backs up certain metadata requests. In fact, responding to every metadata request sent by the client, the MDS sends the desired metadata response with the metadata requests in the Active Request List, which holds certain most recent metadata requests. In fact, every metadata request in the Active Request List includes the original request sent by the client, unique sequential number generated by the MDS, and the relevant stamp. On the other hand, after receiving the reply from the MDS, the client creates an in-memory log to cache the metadata requests sent by the MDS for the possible metadata recovery when the active MDS has crashed in the future. Besides, for the purpose of improving the reliability, each metadata request can be cached by more than one client; thus, the backup metadata request is still available though one of the host clients has failed to resend the backup requests.

**Figure 2:**
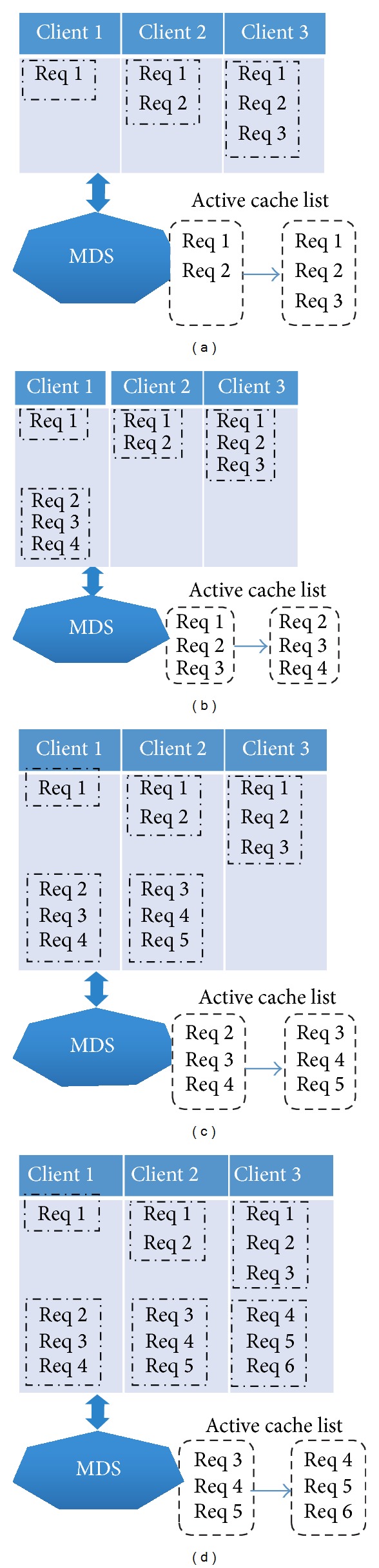
Backing up multiple requests on client side.

We assume that there are 3 clients and they send metadata requests sequentially; besides, the size of Active Request List is configured as 3, which means 3 metadata requests handled by the MDS most recently will be cached on the MDS side. To put it from another angle, there should be 3 metadata requests in each backup log on the client side and each metadata request will be backed up by 3 clients. Without doubts, when the MDS has totally received less than 3 metadata requests, the number of metadata requests in the backup log should be less than 3, as well. Namely, while the number of dead client is less than the size of Active Request List, the MDS can still collect a complete metadata request list resent by other active clients.  Figure 2 shows the instance of caching multiple metadata requests on the client side in detail (the stamp information is ignored in this subsection). In Figure 2(a), all 3 clients have their own backup logs after sending their metadata requests; after* client 1* sends a new metadata request, the MDS first updates the Active Request List and then responds to* client 1* with the corresponding metadata response and the metadata requests in the Active Request List; next* client 1* creates a backup log to record the received metadata requests, which is illustrated in Figure 2(b); finally,* client 1* works as normal, such as parsing the metadata response and communicating with associated storage servers. Figures 2(c) and 2(d), respectively, demonstrate the situations when* client 2* and* client 3* have sent their metadata requests sequentially.

### 2.3. Stamp-Based Metadata Recovery

**Figure 3:**
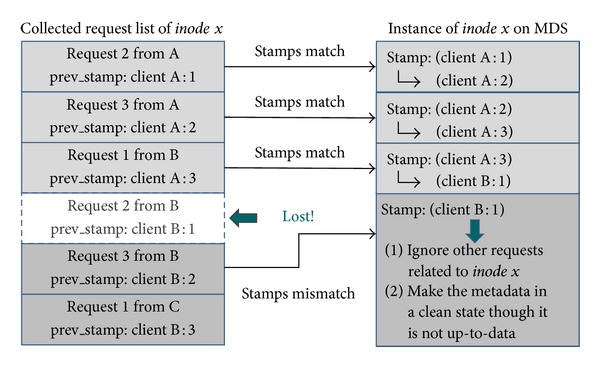
Stamp-based metadata recovery.

A stamp-based metadata recovery is employed by the log-less metadata management mechanism; Figure 3 describes the main idea of stamp-based recovery. In fact, each entry in the collected request list has a metadata request and the previous stamp of the associated* inode*. On the other hand, the MDS first checks whether the stamp of* inode* matches the previous stamp of the collected request or not. If stamps match, the MDS plays the backup request and then updates the stamp of* inode* with the client ID (e.g.,* client A*) and log ID (e.g.,* request 3*) of the last reexecuted metadata request; in case the stamps mismatch, all collected metadata requests associated with the same* inode* will be thrown away from the collected request list for keeping metadata in a clean state though it might not be up-to-date.

### 2.4. Implementation

We have implemented a prototype parallel file system from scratch in C and run it in the Linux environment. The implementation has three modules running at the user level:

- the module of active metadata server, which works for providing metadata service;
- the module of storage server which is responsible for the management of real file data;
- the module of client file system which has been designed and implemented based on FUSE [10].

Since our implementation is a prototype system used for illustrating whether the ideas presented in this paper are feasible or not, for fairness in the comparison experiments, we have also implemented other parallel file systems with different properties (such as the parallel file system that employs log-based metadata management mechanism) based on the source code we have developed as our comparison counterparts.

## 3. Experiments and Evaluation

This section describes the experimental methodology for evaluating our implemented file system and reports the experimental results. First, we introduce the experimental platform for conducting all experiments. Then we show the experimental results related to the overhead associated with backing up metadata requests on the client side. Next, the benefit of log-less metadata management mechanism to metadata processing will be demonstrated and highlighted. Finally, the I/O throughput will be measured and presented.

### 3.1. Experimental Platform and Benchmark

We employed two cluster, which labeled as cluster 1 and cluster 2, to conduct our evaluation experiments. Consequently, one active MDS, 4 storage servers are deployed on the 5 nodes of cluster 1; the client file systems are installed on the 32 nodes of cluster 2; these two clusters are connected by 10 GbE Ethernet. Tables 1 and 2 show the specifications of the nodes on the two clusters. Moreover, the following benchmarks are used in the evaluation experiments.

- mdtest HPC Benchmark 1.8.3 is an MPI-coordinated metadata benchmark. It is the most frequently used benchmark to test the performance of the metadata server under intensive create/stat/remove operations on empty files and directories in parallel file systems [11].
- NAS-BTIO 3.3 is an extension of NAS BT benchmark. It is derived from computational fluid dynamics (CFD) applications and widely used to test the I/O data output capabilities of parallel systems [12]. NAS-BTIO is designed to solve 3D compressible Navier-Stokes equations. Since the access pattern in NAS-BTIO is noncontiguous in memory and in the file, the MPI I/O library is used for its on-disk file access [13].
- MADbench2 is an I/O benchmark derived from a real world application analyzing massive cosmic microwave background radiation in the sky from noisy pixelated datasets from satellites [14]. An extremely large amount of data is written to a disk and then read back from the disk as the calculation progresses. Since MADbench2 performs large, contiguous mixed read and write patterns as matrix operations with a variety of parameters (SHARED or UNIQUE files, POSIX versus MPI I/O, etc.), it has become a popular and often used benchmark in the parallel I/O community [15].

### 3.2. Overhead of Backing up Requests on Client

For the purpose of metadata recovery, the log-less metadata management mechanism adopts backing up sent metadata requests on the client side; once the former active MDS has crashed, the clients will then resend the logged, uncommitted metadata requests to the rebooted MDS or the standby MDS for restoring the lost metadata. In order to investigate the overhead due to backing up requests on the client side in our mechanism (such as making backup log records), we chose a benchmark, which simply copies an empty file 10000 times per minute. Each copy operation contains several metadata requests. Equation (1) shows the components of the metadata operations in detail:

(1) 1  copy=1  getattr+3  lookups+1  mkmod+2  opens+1  read.

For each metadata operation, the client should make a corresponding backup metadata request. Thus, the client should keep a lot of backup metadata requests after the copy operations. We should mention that there is no* write* metadata operation in (1), because the source file is an empty file. In the case of reading 0 bytes from the source file, our implemented system does not write any data to the newly created file.

**Figure 4:**
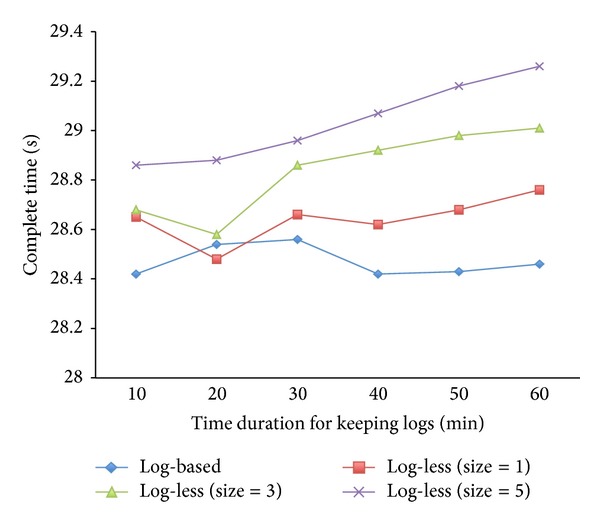
The overhead of backing up requests on client side.

Figure 4 shows the average execution times on both the log-based metadata management mechanism and log-less metadata management mechanisms (with different sizes of Active Request List, which means each log created by the client should include different number of backup metadata requests) for copying 10000 file per minute, which has been repeated 60 times in sequence (i.e., 60 minutes running time). The *x*-axis indicates the duration of time for which metadata requests are kept on the client side before committing the metadata changes to the disk on the MDS side. After that, the corresponding logs kept by the clients can be released. It is obvious that keeping logs on the client side brings about no more than 2.9% overhead other than for certain memory space needed for storing the backed up logs temporarily.

Moreover, it is clear that more time and more memory space are consumed while the size of Active Request List is becoming larger, that is because the more requests should be included in each client log. On the other hand, the larger size of Active Request List means much more reliability; it can tolerate more crashes of clients or loss of the backup logs. Since the size of Active Request List is configurable, it is not difficult to balance the reliability and performance overhead with the agreeable size of Active Request List.

### 3.3. Metadata Processing

To improve the metadata processing throughput, all metadata is kept in the memory of metadata server in the implemented file system. As a matter of fact, metadata performance is critical to the whole file system; this information has been used to find bottlenecks in the important area of metadata processing as a growing file system performance issue. The mdtest benchmark [11] was used to test the metadata performance of our implemented prototype file system, and we measured metadata performance with various clients from 1 to 16. In the experiments, we configured one task per client node, and every task executed the following command:* mdtest -u -d/mnt/pfs/newfs/temp/-b 3 -z 5 -I 100 -i 3*.

**Figure 5:**
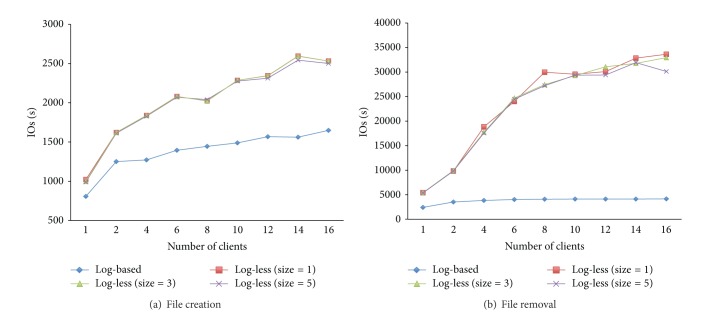
Metadata performance: creation operation.

Figures 5(a) and 5(b) show the results when clients created and deleted objects (i.e., files), respectively. In these figures, *x*-axis represents the number of clients involved in the tests, and *y*-axis denotes the number of completed I/O operations per second (called IOPS, higher is better). From the results reported in Figures 5(a) and 5(b), it is safe to conclude that log-less mechanisms with different sizes of Active Request List outperform the log-based mechanism; namely, in contrast to the conventional metadata management mechanism, the newly proposed log-less mechanism can improve the speed of metadata processing, which is critical to metadata-intensive applications. In addition, the log-less mechanism with larger size of Active Request List performs a little worse than the mechanism with smaller size of Active Request List since more metadata requests should be handled.

### 3.4. I/O Throughput

**Figure 6:**
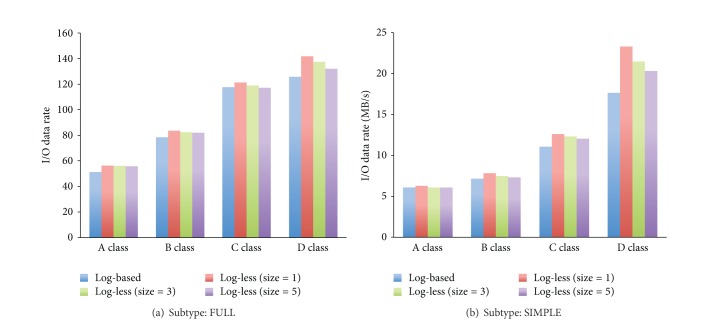
The results of BTIO benchmark.

We also selected BTIO benchmark and madbench2 benchmark to measure the I/O data rate of the file systems with different properties. Figures 6(a) and 6(b) show the results of BTIO benchmark while the subtype is FULL and SIMPLE, respectively. It is clear that the log-less mechanism can obtain more I/O data rate than the log-based mechanism, for example, more than 15% improvement, while the filetype is SIMPLE and the subclass is D, which is shown in Figure 6(b). The reason for the less data throughput when adopting Log-based mechanism is due to making logs to nonvolatile storage on the MDS must cause negative effect on I/O data throughput. In addition, the log-less mechanism with smaller size of Active Request List outperforms the log-less mechanism with larger size of Active Request List because both clients and the MDS just need to process less backup metadata requests.

**Figure 7:**
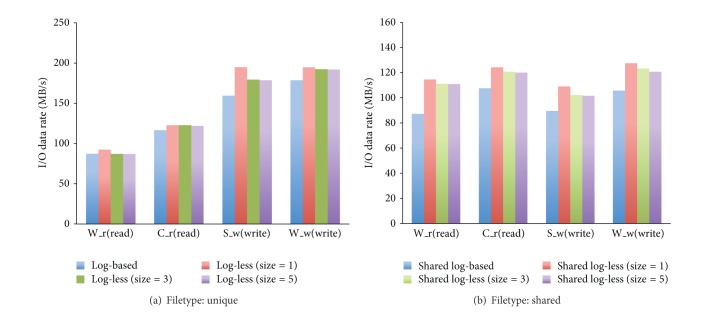
MADbench2 results: IOMETHOD = MPI, SYNC, 18KPIX, 16BIN.

Figures 7(a) and 7(b) demonstrate the experimental results of MADbench2 with unique and shared filetype, respectively. As a matter of fact, the results have similar trend to that of BTIO. From the experimental results presented in the section, we can safely make a brief summary that log-less metadata management mechanism on the MDS can improve not only the speed of metadata processing, but also the I/O data throughput.

## 4. Related Work

In this section, we will outline several metadata management mechanisms for restoring lost metadata updates in the conventional parallel file systems.

- *Logging Metadata Updates.* The traditional logging metadata updates mean every log should be flushed to nonvolatile storage before responding to the client requests. This mechanism is quite straightforward and can ensure metadata consistency correctly, but it may result in quite large overhead for I/O operations, and then impose negative effect on normal metadata processing. As a matter of fact, a major part of conventional distributed and parallel file systems, such as the Gfarm file system [5] and the Google file system [7], employs this kind of mechanism to ensure the metadata consistency even though the former MDS has crashed unexpectedly.
- *Soft Updates.* The soft updates mechanism tracks dependencies among changes to cached (i.e., in-memory) copies of metadata and enforces these dependencies, via update sequencing, as the dirty metadata blocks are written back to nonvolatile storage [16]. Compared with the mechanism of logging metadata updates, the mechanism of soft update can yield performance improvements for metadata-update-intensive applications; however, since certain changes are cached in the memory, metadata consistency cannot be ensured affirmatively once the MDS goes into nonoperational state.
- *Synchronous Metadata Replication.* With the mechanism of synchronous metadata replication, all metadata changes are replicated in the standby MDS before the active MDS responds to the clients. Wang et al. [17] have designed and implemented a hot standby replication mechanism in the Hadoop file system [18] to provide a highly available metadata service. This replication mechanism incurs around three times the delay in metadata responses. In case the active MDS crashes, the hot standby MDS replaces the failed one and continues to provide metadata service for the outside clients based on its current state, without any lost metadata changes. However, hot replication model results in the latency in the responses to the clients, because every metadata response cannot be delivered until the corresponding metadata change has been replicated to the standby one. Consequently, it incurs performance degradation of I/O data throughput definitely.

## 5. Concluding Remarks

This paper has proposed a novel metadata management scheme on the metadata server for distributed and parallel file systems. In this newly proposed log-less metadata management mechanism, all client file systems back up the sent metadata requests till the associated metadata changes have been committed to the nonvolatile storage by the active MDS, and the evaluation experiments show that backing up metadata requests on the involved clients only results in no more than 2.9% overhead. On the other hand, the log-less mechanism makes the MDS freed from logging metadata changes to nonvolatile storage systems; thus, compared with conventional log-based metadata management mechanisms, the speed of metadata processing and I/O data throughput can be improved significantly. As a matter of fact, the log-less metadata mechanism presented in this paper can be also applied to other conventional parallel file systems such as the PVFS file system [19] and the Hadoop file system, or their extensions, as well.

## Figures and Tables

**Table 1:** Specification of nodes on cluster 1.

CPU	2 **×** Intel Xeon E5502 1.86 GHz
Memory	6 **×** 4 GB 1066 MHz/DDR3 Memory
Disk storage for MDS	3∗160 GB 7200 rpm SATA HDD
Disk storage for OST	5∗160 GB 7200 rpm SATA HDD
Network	Intel 82598EB, 10 GbE Ethernet
Operating system	Debian GNU/Linux 5 (Kernel 2.6.27)

**Table 2:** Specification of nodes on cluster 2.

CPU	AMD Quad-Core Opteron 8356 2.3 GHz
Memory	32 GB 1066 MHz/DDR3 Memory
Local disk storage	250 GB 7200 rpm SATA HDD
Network	IP over Myrinet
Operating system	Centos 5.1 (Kernel 2.6.18)
